# Effects of Behaviour Change Interventions Combined With Exercise on Device‐Measured Physical Activity in People With Hip and Knee Osteoarthritis: A Systematic Review and Meta‐Analysis

**DOI:** 10.1155/jare/5852133

**Published:** 2026-08-17

**Authors:** Rebecca M. Meiring, Mitchell Campbell, Abhishta Sood, Daniel O’Brien, Estelle D. Watson

**Affiliations:** ^1^ School of Exercise, Sport and Rehabilitation Sciences, Faculty of Science, University of Auckland, Auckland, New Zealand, auckland.ac.nz; ^2^ School of Physiology, Faculty of Health Science, University of the Witwatersrand, Johannesburg, South Africa, wits.ac.za; ^3^ Department of Physiotherapy, School of Clinical Sciences, Active Living and Rehabilitation Aotearoa New Zealand, Health and Rehabilitation Research Institute, Auckland University of Technology, Auckland, New Zealand, aut.ac.nz

**Keywords:** ageing, behavioural therapy, motivational interviewing, physical activity, sedentary behaviour

## Abstract

Behaviour change techniques (BCTs) are recommended as core components of physical activity (PA) interventions for people with lower limb osteoarthritis (OA), and identifying effective BCTs is an emerging research priority. This systematic review aimed to evaluate the effects of combined behaviour change and exercise interventions on objectively measured total PA (primary outcome) and other device‐measured activity behaviours, including moderate‐to‐vigorous physical activity (MVPA), steps and sedentary behaviour, in people with hip or knee OA (secondary outcomes). Five databases were searched from inception until September 2025. The titles of identified randomised controlled trials (*n* = 907) were screened by two reviewers, and 12 studies involving 1162 participants with OA were included. Study quality was assessed using the SIGN checklist, and certainty of evidence was evaluated using the GRADE approach. Most studies were rated as low quality, and the certainty of evidence was low to very low across all outcomes. Random‐effects meta‐analyses found little to no difference for the use of behaviour change and exercise interventions for our primary outcome of total PA postintervention (3 studies, *n* = 246; MD 21.25 min/day, 95% CI ‐26.11–68.61) or at 12‐month follow‐up (3 studies, *n* = 221; MD 30.52 min/day, 95% CI ‐25.42–86.45). A meta‐analysis of four studies (*n* = 307) found a small increase in device‐measured MVPA at short‐term follow‐up (MD 2.53 min/day, 95% CI 0.18 to 4.88; *I* ^2^ = 21%). No significant effects were observed for any of the other outcomes (long‐term MVPA, steps or sedentary behaviour). The existing evidence is limited, and findings should be interpreted cautiously given the low to very low certainty of the evidence and the small number of studies available. This highlights the need for higher quality studies with clear BCT reporting and fidelity procedures to establish whether behaviour change combined with exercise improves PA behaviours.

## 1. Introduction

Osteoarthritis (OA) is a prevalent condition, affecting approximately 8% of the global population and making up a large proportion of all musculoskeletal causes of disability [1]. Increasing pain and a decline in functional mobility are commonly experienced with disease progression, particularly with weight‐bearing activities [2]. In people with knee and hip OA, being more physically active and spending less time in sedentary behaviour (SB) is associated with improved physical function and quality of life, a reduction in pain and maintenance of functional independence [3–7]. As a result, the management of OA early in the disease process focuses on exercise and lifestyle modifications, with the most recent recommendations including adapted physical activity (PA) as a core treatment [8].

Engaging in PA can be challenging for people living with OA. The potential barriers to participation in regular PA are pain, disability due to physical restrictions, lack of knowledge about the efficacy of PA, previous negative experiences with activity, lack of motivation, discipline and behavioural habits of a sedentary lifestyle [9]. As a result, a significant proportion of people with OA are considered insufficiently active [2, 10] and spend a large proportion of their day in SB [11, 12]. There is also evidence that even following joint replacement surgery, SB does not change significantly [11, 13]. Hence, targeted behaviour interventions are required to promote, facilitate and maintain behaviour change to increase PA levels and reduce SB in people with OA.

Behaviour change techniques (BCTs) are coordinated strategies employed to elicit a positive change in specified health behaviours [14]. In the context of PA and SB, motivational interviewing (MI) and cognitive behavioural therapy (CBT) are two examples of BCTs used to correct erroneous beliefs and behaviours that prevent an individual from engaging in PA [15, 16]. Using MI in combination with usual care has been shown to lead to modest improvement in PA levels [15], even in those living with chronic health conditions [18]. To date, only a small number of reviews have examined how BCTs have been integrated into exercise programmes for people with hip and knee OA. For example, a meta‐analysis by Pitsilledes et al. [16] reported that CBT delivered in combination with exercise results in a reduction of knee pain reported via the WOMAC pain subscale. Furthermore, in another meta‐analysis, the use of BCTs effectively promoted self‐reported adherence to PA in the short and long term for people with lower limb OA [19]. Recent recommendations also emphasise that PA interventions for people with OA should incorporate BCTs to support long‐term adherence, reflecting growing consensus that BCTs are essential components of effective PA promotion in this population [20, 21].

Within the last decade, technological advancements have led to a surge in the popularity of wearable technology and digital devices, opening a new avenue for practitioners and researchers to monitor patient adherence to and levels of PA and SB [22]. Device‐based PA and SB measurement using accelerometers is considered valid and often more reliable than self‐report measures because people, particularly older adults, are susceptible to recall bias [23, 24] and overestimating PA [10]. Activity tracking devices can also provide exercise feedback in real‐time, reinforce and impose positive activity behaviours through push notifications and bypass memory problems, all of which are pertinent for the older adult population [25, 26].

Because of the clear benefits of engaging in PA for overall health in people with OA and the evidence that the application of BCTs has improved engagement in PA in other populations, we aimed to synthesise and summarise the evidence on the effects of combined BCT and exercise interventions, compared to usual care or exercise support alone, on device‐measured PA behaviours in people living with OA. The primary objective was to determine whether BCTs combined with exercise improve objectively measured total PA compared with control conditions. Secondary objectives were to examine effects on other device‐measured activity behaviours, including moderate‐to‐vigorous physical activity (MVPA), step counts and SB.

## 2. Materials and Methods

This systematic review and meta‐analysis was conducted and reported in accordance with the Preferred Reporting Items for Systematic Reviews and Meta‐Analysis (PRISMA) guidelines [27] (Supporting File 2: PRISMA Checklist). This review was registered with the international database for prospective register of systematic reviews (PROSPERO ID: CRD42022318733); a protocol is not available.

### 2.1. Search Strategy and Selection Criteria

A systematic search was conducted in PubMed, MEDLINE (Ovid), SPORTDiscus, the Cochrane Library and CINAHL from inception until September 2025. Searches combined terms related to behaviour change interventions (e.g. behaviour change, cognitive behavioural therapy, CBT, motivational interviewing), physical activity (e.g. physical activity, device‐measured physical activity, accelerometer, accelerometery) and osteoarthritis. Search terms were adapted as required for the indexing systems and search interfaces of individual databases. The core search concepts were combined using Boolean operators (AND/OR) (Table 1).

**TABLE 1:** Search terms and Boolean operators.

Search terms and operators	
Search strategy	((“behavio^∗^ change” OR “behaviour change” OR “behavior change” OR “cognitive behavioural therapy” OR “cognitive behavioural therapy” OR CBT OR “motivational interview^∗^” OR MI) AND (“physical activity” OR PA OR “device‐measured physical activity” OR accelerometer^∗^ OR accelerometr^∗^) AND (osteoarthritis OR OA))

Studies were included if (1) participants were community‐dwelling adults with a clinical diagnosis of hip/knee OA and had radiological evidence of OA; (2) published randomised controlled trials (RCTs) that employed BCTs as primary means to increase PA in osteoarthritic populations compared to a control group (either usual care, exercise or intervention that did not receive BCTs). Studies were excluded if participants had additional pathologic conditions that could affect their PA (such as fractures, surgery or neurological conditions) or were not printed in English.

### 2.2. Study Selection and Data Extraction

Once study duplicates were removed, the remaining titles and abstracts were independently screened for eligibility by two authors (EW and RM), and any disagreement was resolved by discussion and consensus. Data were extracted for objectively measured PA outcomes. The primary outcome was total PA (min/day). Secondary outcomes included MVPA (min/day), steps/day, SB (min/day) and other device‐measured PA outcomes. Data were collected for all reported data collection time points and grouped for synthesis according to outcome measure and time point (e.g. postintervention and follow‐up). Where multiple follow‐up time points were reported, data were pooled separately. Data were extracted using custom‐designed Excel spreadsheets. Data extracted included study design, participant characteristics, method and means of cognitive/behavioural change utilised, device type and placement location, duration of device data collection and outcome raw data. Any discrepancies in extracted data were resolved by consensus, or where consensus could not be reached, a third researcher made the final decision. Quality of study methods was assessed using Methodology Checklist 2 for RCTs from the Scottish Intercollegiate Guidelines Network [28].

### 2.3. Statistical Analysis

Meta‐analysis was undertaken using Review Manager (Version 5.4.1; The Cochrane Collaboration, 2024. Available at https://revman.cochrane.org) using a random‐effects model. Meta‐analysis results were visually displayed using forest plots and reported as pooled mean differences (MDs) with 95% confidence intervals (CIs). The MD with 95% CIs was calculated for continuous outcomes. For studies reporting medians with ranges, interquartile ranges or 95% CIs, the means and standard deviations were estimated to enable inclusion in the meta‐analysis [29]. Effect sizes were allocated as follows: > 0.8, a large effect; 0.5 to 0.79, a moderate effect; and 0.2 to 0.49, a weak effect [30]. The variability in effect estimates due to heterogeneity between studies was assessed using the *I* ^2^ statistic, where < 25% indicates low risk of heterogeneity, 25%–75% indicates moderate risk, and > 75% indicates high risk of heterogeneity [31]. Subgroup and sensitivity analyses were not performed because only 2–3 studies contributed to each pooled outcome, limiting the ability to explore sources of heterogeneity or assess the robustness of the results. Publication bias based on funnel plots was only used if more than 10 studies were included in the analysis. Direction of findings tables were used to summarise the evidence if meta‐analysis was not possible.

## 3. Results

### 3.1. Search Results

**FIGURE 1:**
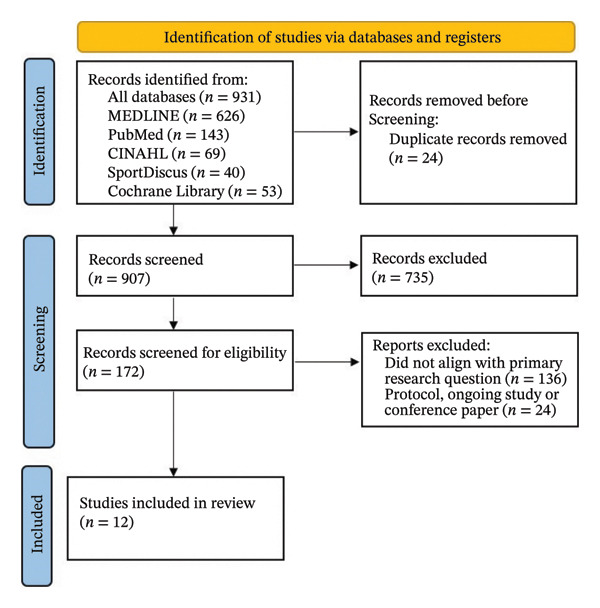
PRISMA flow diagram of search process.

The searches across all included databases identified 907 studies. Studies that appeared in duplicate (*n* = 24) were removed. The titles of studies were screened, and *n* = 735 were removed after not meeting the criteria. The abstracts of the remaining 172 articles were assessed for eligibility using the predetermined inclusion and exclusion criteria. This process resulted in a further 160 studies being omitted. Hence, twelve studies were retained for critical appraisal and data extraction (Figure 1).

### 3.2. Characteristics of Included Studies

Study characteristics and data are summarised in Table 2. All studies were done in high‐income countries, with 8 conducted in the United States, 3 in Australia and one in Canada. Sample sizes ranged from 31 to 193 participants. The mean age of participants across all the studies was 64.6 years old, with the mean ages ranging from 59.6 to 75.3 years old. Each study included males and females, but most had more female participants. Eight studies included participants with knee OA only, one study included participants with knee and/or hip OA, and three studies included participants with either hip or knee OA. Some studies provided further detail around the specifics of the participants’ OA, with Gilbert et al. [32] reporting on mean disease duration and three studies reporting on the diagnostic characteristics of participants’ OA using the Kellgren–Lawrence (KL) scale [23, 33]. All studies reported mean participant BMI, with scores ranging from 27.6 to 33.7, with an average BMI of 30.9 across all twelve studies.

**TABLE 2:** Study characteristics and data.

Author (year)	Location	Participants	Participants, *n*	Device used	Wear position and time	Objective PA outcomes	Intervention/control	Fidelity of the intervention assessed	Length of the intervention/follow‐up
Bell et al. [34]	Melbourne, Victoria, Australia	10/22 (M/F) 71yo (mean age) BMI: 30.5 (mean) OA as determined by clinical diagnosis according to NICE guidelines as (1) being aged > 45y; (2) activity‐related knee pain; (3) morning stiffness that lasts < 30 min or no stiffness Region: knee OA	31 (IG:15; CG:17)	ActivPAL accelerometer	Right thigh; 7 days	Average daily steps; minutes with cadence > 100 steps per minute; minutes where bouts were > 1 min in duration/day	IG: Motivational interviewing (5 × 30‐min sessions over 10 weeks) and digital support tool (website with information about PA, knee OA, goal setting, research and activities, patient stories). CG: Usual care, permitted to engage in routine services for knee OA management including visits to GP, physiotherapist and other health professionals	Yes, researcher received training in MI and was graded proficient according to the MITI.	10 weeks; FU: 3 months

Bennell et al. [35]	Melbourne, Australia	62/106 (M/F) 62.3yo (mean age) BMI: 31.5 (mean) American College of Rheumatology clinical criteria for knee OA Average knee pain > 4 (NPRS) Region: knee OA	168 (intervention = 84; control = 84)	ActivPAL accelerometer	Anterior thigh, 7 consecutive days	Time stepping (hrs/day); steps (n/day)	IG + CG: 5 × 30‐min PT‐led education and exercise sessions. IG only: 6 – 12 telephone‐delivered CBT and MI‐directed coaching sessions.	Yes, 10% of coaching sessions were randomly selected and rated for HealthChange practice principles using 11‐point NRS.	6 months FU: 12 months; 18 months

Bossen et al. [36]	Melbourne and Brisbane, Australia (Multisite)	70/129 (M/F) 62yo (mean age) BMI: 27.6 (mean) KOA (63%) Self‐reported OA and inactivity (30 min of moderate PA < 5 × p/w) Region: knee and/or hip OA	199 (intervention = 100; control = 99)	ActiGraph GT3X accelerometer	Waist, 5 consecutive days	Total PA (min/day); SB (min/day)	IG group received Join2move web‐based intervention. CG: Waitlist control group	Unspecified.	9 weeks FU: 3 months; 12 months

Christiansen et al., [37]	Colorado, USA	81/3 (M/F) 67yo (mean age) BMI: 29.1 (mean) OA as determined by the schedule for unilateral TKA Region: knee OA	92 (IG:46; CG:46)	ActivPAL micro‐accelerometer; Fitbit wrist‐worn sensor	1/3 of the distance between the hip and the knee; 10‐day wear period	Daily average step count	IG: 1:1 telerehabilitation (10 × 30‐min sessions over 12 weeks) including participant‐tailored action plans with functional and stepping goals, education, self‐monitoring, feedback, barrier and facilitator identification, problem‐solving, action planning, encouragement. CG: 1:1 telerehabilitation (10 × 30‐min sessions over 12 weeks) focused on health‐related education unrelated to PA (pain management, diet, medication management, home safety, falls and fractures).	Yes, monthly fidelity reviews using checklists, direct observation, review of treatment notes, Fitbit usage monitored.	12 weeks; FU: 38 weeks

Focht et al. [38]	Ohio, USA	4/36 (M/F) 63.5yo (mean age) BMI: 32.7 KL scale stage 1 or 2 Self‐reported knee pain on most days of the month Difficulty with ADLs due to pain < 20 min of structured PA per week Region: knee OA	80 (intervention = 40; control = 40)	Lifecorder Plus accelerometer	Waist: right hip, 7 consecutive days	Total PA (min/wk); MVPA (min/wk)	IG: 1‐h exercise + 20‐min group‐based CBT counselling CG: Supervised exercise: 3 ×/wk, 1‐h sessions	Unspecified	IG: 9 months; CG: 3 months FU: 12 months

Gilbert et al., [39]	Chicago, USA	62/93 (M/F) 63.1yo (mean age) BMI: 31.3 (mean) Mean disease duration: 10.9 years Region: knee OA	185 (intervention = 93; control = 92)	ActiGraph GT1M accelerometer	Waist: right hip; 7 consecutive days	Total activity (min/day); MVPA (min/day)	IG + CG: Brief physician PA advice session to meet the recommended guidelines. IG: 3 sessions of MI counselling in first year; 2 sessions in second year.	Unspecified	24 months

Gillcrist et al., [38]	Philadelphia, USA	28/3 (M/F) 59.6yo (mean age) BMI: 33.7 (mean) OA as diagnosed by a physician; radiographic evidence (Kellgren–Lawrence grade ≤ 1) Region: knee OA	31 (IG:16; CG:15)	Fitbit tracker		Daily and 2‐week average step count	IG + CG: Personalised daily step goal based on baseline step count. IG: Social support partner who received updates on progress and served as encouragement. Game‐based intervention where they received 70 points in the beginning of the week and lost points should they not meet their goal. If they met their goal, they could advance through levels and earn ‘medals’. CG: Received weekly updates.	Unspecified	32 weeks

Li et al., [40]	Vancouver, Canada	9/42 (M/F) 64.9yo (mean age) BMI: 29.4 (mean) Diagnosed with OA (*n* = 37 or met OA criteria (*n* = 14) Region: knee OA	51 (intervention = 26; control = 25)	SenseWear Mini triaxial accelerometer	Triceps; 7 consecutive days	MVPA (min/day); purposeful activity (min/day); steps (n/day); SB (min/day)	IG: Education, Fitbit Flex‐2, access to FitViz app, 4 × biweekly PT phone call. CG: Monthly newsletter and included in IG at week 14.	Intervention fidelity was tracked on (1) adverse events, (2) adherence and (3) Fitbit wear. 81% of all participants met all 3 criteria.	8 weeks. FU: 13; 26 and 39 weeks

Murphy et al., [41]	Michigan, USA	11/35 (M/F) 63.5yo (mean age) BMI: 31.9 (mean) Reported knee pain > 3/10 on NPRS of a > 3‐month duration Region: knee OA	46 (intervention = 31; control = 15)	Actiwatch‐S accelerometer	Wrist; 7 consecutive days	Average weekly activity (counts/min)	IG: 8 × weekly 1‐h in‐person sessions, with modules and homework sheets. CG: Usual OA care	Unspecified. Therapists were trained and followed a manual.	6 weeks FU: 6 months

Murphy et al., [42]	Michigan, USA	74/119 (M/F) 64.7yo (mean age) BMI: 31 (mean) Radiographic evidence of knee or hip OA (> 2 KL scale) Region: knee or hip OA	193 (tailored = 64; general = 66; control = 63)	Actiwatch‐S accelerometer	Wrist; 7 consecutive days	Average weekly PA (counts/min); PA variability (SD of activity counts)	The tailored and general IG: 3 × individual in‐person sessions every 7–10 days. Tailored: Individualised summary report. CG: Usual OA care	Therapists logged treatment time and checklist of items to cover. 10% of randomly selected audiotaped sessions met all criteria.	30 days. FU: 10 weeks; 6 months

Murphy et al., [43]	Michigan, USA	8/24 (M/F) 61.9yo (mean age) BMI: 32.3 (mean) Region: knee or hip OA	32 (IG tailored = 17; CG:15)	Actiwatch‐S accelerometer	Nondominant wrist, 5 days	Total physical activity (average counts/day); peak physical activity (counts)	IG + CG: 2 × sessions over 2 weeks, education module IG: Tailored individualised information around activity pacing and goal setting.	Unspecified	2 weeks FU:10 weeks

Murphy et al., [44]	Michigan, USA	6/48 (M/F) 75.3yo (mean age) BMI: 30.1 (mean) OA as determined by American College of Rheumatology clinical criteria Region: knee or hip OA	54 (IG:28; CG:26)	Actiwatch‐S, Mini‐Mitter	Wrist; 3 consecutive days	Average weekly PA (counts/min); average weekly peak PA (counts/min)	IG: Exercise and activity strategy training (2 × 1.5 h per week for 4‐week group sessions, 1 individualised session at home), 2 × sessions over the next 6 months CG: Exercise programme and education sessions (2 × 1.5 h per week for 4‐week group sessions), 2 × sessions over the next 6 months	Unspecified. Treatment time was controlled to improve fidelity.	4 weeks; FU: 6 weeks

*Note:* yo = years of age, KOA = knee osteoarthritis, OA = osteoarthritis.

Abbreviations: BMI = body mass index, CG = control group, FU = follow‐up, GP = general practitioner, IG = intervention group, KL = Kellgren and Lawrence, M/F = male/female, MI = motivational interviewing, MITI = Motivational Interviewing Treatment Integrity, NPRS = numerical pain rating scale, PA = physical activity, SD = standard deviation, TKA = total knee arthroscopy.

All included studies used various accelerometers to objectively measure PA (ActiGraph, ActivPAL, Actiwatch‐S, Lifecorder EX Plus and Fitbit SenseWear Mini). There was some variation in wear time duration between the studies. A period of seven consecutive days was used in all but five of the studies, with Bossen et al. [36] and Murphy et al. [41] making use of 5 days of accelerometry time; Murphy et al. [42] employing a 3‐day period and Christiansen et al. [37] using a 10‐day wear period. Gillcrist et al. [38] used a Fitbit tracker which participants wore (location unspecified) for the duration of the study (32 weeks). Positioning of the accelerometer also varied between studies. In three studies [34, 35, 37], participants wore the accelerometer on the anterior thigh, another on the triceps [39], three studies asked participants to wear the accelerometer on the waist [23, 32, 36], and the rest wore it on the wrist [33, 40–42].

The duration of each intervention ranged from 30 days to 24 months and consisted of a combination of BCTs and exercise therapy. Five study interventions were underpinned by CBT or MI theories. In contrast, one study was directed by group dynamics and social cognitive theory [23], two studies incorporated operant theory‐driven activity pacing interventions [33, 41], and one study had an intervention guided and inspired by a pre‐existing behaviour graded activity (BGA) programme [36]. Murphy et al. [42] based their intervention on an activity strategy training (AST) programme; Bell et al. [34] used several BCTs to encourage participant’s self‐management; and Gillcrist et al. [38] used game‐play and social incentivisation. Most studies used in‐person sessions [23, 33, 40, 41], online [36] or telephone‐based, or a combination [32, 35, 37–39]. Sessions varied between individual [32–35, 37–41], group‐based [23] or a combination [42].

Across the included studies, six reported implementing procedures to support or assess intervention fidelity. These approaches included formal training in MI with proficiency checks [34], random sampling of coaching sessions for fidelity scoring [35], monthly fidelity reviews using checklists and observation [37], monitoring of Fitbit wear and adherence [37, 39], therapist training supported by manuals [40], treatment logs and review of audiotaped sessions [33]. One study also controlled treatment time to enhance fidelity [42]. The remaining six studies did not report any fidelity procedures [23, 32, 36, 38, 41].

### 3.3. Primary Outcome

#### 3.3.1. Total PA

**FIGURE 2:**
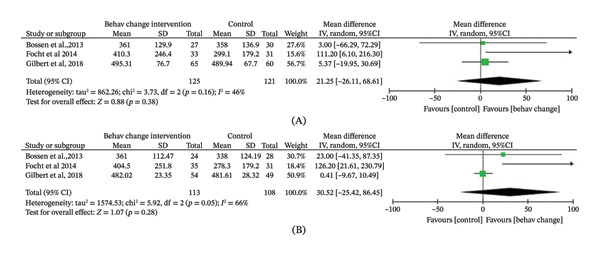
Forest plot comparison for total PA (min/day) at (A) 10–13 weeks and (B) 12 months of follow‐up.

Evidence from three studies [23, 32, 36] suggests that behaviour change interventions may result in little to no difference in total PA (minutes per day) postintervention (Figure 2A; 246 participants, MD: 21.25, 95%CI: −26.11 to 68.61, *p* = 0.38, *I* ^2^ = 46%) and at 12‐month follow‐up (Figure 2B; 221 participants, MD: 30.52, 95%CI: −25.42 to 86.45, *p* = 0.28, *I* ^2^ = 66%).

### 3.4. Secondary Outcomes

#### 3.4.1. MVPA

**FIGURE 3:**
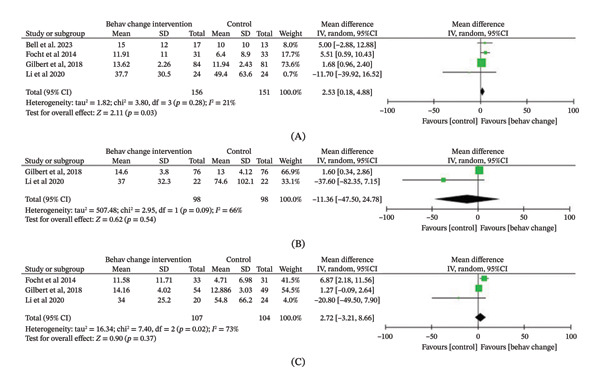
Forest plot comparison for MVPA (min/day) at (A) 10–13 weeks, (B) 6 months and (C) 9–12 months of follow‐up.

Results from four studies [23, 32, 34, 39] and 307 participants suggest that behavioural change interventions may result in a small increase in device‐measured MVPA (minutes per day) in the short term (10–13 weeks) (Figure 3A; MD: 2.53, 95%CI: 0.18 to 4.88, *p* = 0.03, *I* ^2^ = 21%). Results from two studies [32, 39] and 196 participants suggest that behavioural change interventions may result in little to no difference in MVPA (minutes per day) after 6 months (Figure 3B; MD: −11.36, 95%CI: −47.50 to 24.78, *p* = 0.54, *I* ^2^ = 66%). Three studies [23, 32, 39] and 211 participants suggest that behavioural change interventions may result in little to no difference in MVPA (minutes per day) at 9–12 months (Figure 3C; MD: 2.72, 95%CI: −3.21 to 8.66, *p* = 0.37, *I* ^2^ = 73%).

#### 3.4.2. Steps

**FIGURE 4:**
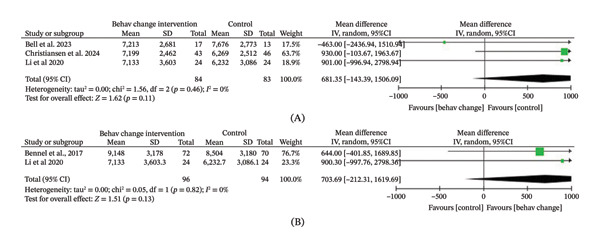
Forest plot comparison for number of steps per day (A) 12–14 weeks of follow‐up and (B) at 3–6 months of follow‐up.

Evidence from 3 studies [34, 37, 39] suggests that behaviour change interventions result in little to no difference in steps per day at 12–14 weeks postintervention (Figure 4A; 167 participants, MD: 681.35, 95%CI: −143.39 to 1506.09, *p* = 0.11, *I* ^2^ = 0%). Evidence from 2 studies [35, 39] suggests that behaviour change interventions may result in little to no difference in steps per day at 3–6 months postintervention (Figure 4B; 190 participants, MD: 703.69, 95%CI: −212.31 to 1619.69, *p* = 0.13, *I* ^2^ = 0%).

#### 3.4.3. SB

**FIGURE 5:**
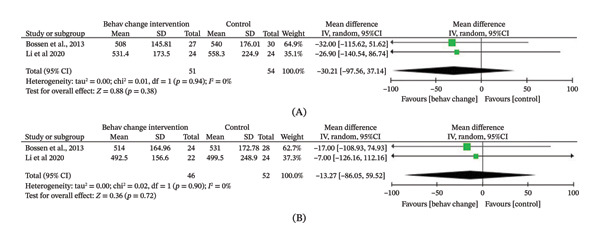
Forest plot comparison for SB (min/day) at (A) 10–13 weeks of follow‐up and (B) at 6–12 months of follow‐up.

Evidence from two studies [36, 39] suggests that behaviour change interventions may result in little to no difference in SB (minutes per day) postintervention (Figure 5A; 105 participants, MD: −30.21, 95%CI: −97.56 to 37.14, *p* = 0.38, *I* ^2^ = 0%). At 6–12 months of follow‐up, data from two studies [36, 39] suggest that behaviour change interventions may result in little to no difference in the amount of time spent in SB (minutes per day) (Figure 5B; 98 participants, MD: −13.27, 95%CI: −86.05 to 59.52, *p* = 0.72, *I* ^2^ = 0%).

#### 3.4.4. Other Objective Measures of PA (Study Defined)

Seven studies [32, 33, 35, 39–42] reported the effects of behaviour change on objectively measured PA, but the methods of assessment and outcome reporting differed between the studies, making it difficult to conduct a meta‐analysis. All seven studies reported no significant difference between intervention and control for all outcome measures (stepping hours per day, total daily activity, peak activity and PA variation), as shown in Table 3.

**TABLE 3:** Summary of other objectively measured physical activity outcomes.

Increase in objectively measured activity	No significant difference in objectively measured activity	Reduction in objectively measured activity
No studies found an increase in objectively measured PA	Bennell et al. [35] found no significant difference in time stepping (hours per day) between intervention (*n* = 72) and control groups (*n* = 70) (2.0 ± 0.7 vs 1.9 ± 0.7).	No studies found a reduction in objectively measured PA
	Gilbert et al. [32] found no significant difference between intervention and control for overall (3, 6, 12 and 24 months of follow‐up) average daily activity minutes (494.11, 95% CI: 514.20 to 474.03 vs 480.11, 95%CI: 497.08 to 463.14, *p* = 0.29).	
	Li et al. [25] found no significant difference in intervention vs control group for time in purposeful activity^†^ (min) (MD: 1.6, 95% CI: −3.0 to 6.1, *p* = 0.50).	
	Murphy et al. [40] found no significant difference between intervention and control groups for average activity (counts/min) (MD = −41.0, 95% CI: −103.19 to 21.19, *p* = 0.19.	
	Murphy et al. [42] found no difference between the three groups for average physical activity (counts/min) (*p* = 0.07) and activity variability (*p* = 0.34)	
	Murphy et al. [41] found no significant difference between intervention and control groups for total physical activity (average counts/day) (*p* = 0.71) and peak physical activity (activity counts) (*p* = 0.71)	
	Murphy et al. [33] found no significant difference between intervention and control groups for peak physical activity (counts) (*p* = 0.69) and total physical activity (counts) (*p* = 0.87)	
	Bell et al. [34] found no significant difference between the intervention and control groups for daily stepping time (MD: 7, CI: −19 to 6; *p* = 0.30), daily time with stepping cadence > 100spm (MD: 5, CI: −0.4 to 11, *p* = 0.67) and daily time with bouts of > 1 min (MD:8, CI: −6 to 21, *p* = 0.27).	
	Gillcrist et al. [38] showed no significant difference between the intervention and control groups for daily step count (2‐week average) at follow‐up (MD:1119, CI:‐562 to 2799, *p* = 0.19).	

^†^Purposeful activity was defined as activity performed at ≥ 4 METs and in bouts ≥ 10 min.

#### 3.4.5. Certainty of Evidence (GRADE Assessment)

Study quality was assessed using the SIGN checklist, and the certainty of evidence for each outcome was evaluated using the GRADE approach. SIGN ratings informed the risk‐of‐bias domain within GRADE, and both assessments were used to interpret the strength and reliability of the findings. The SIGN quality assessment of the studies selected in this systematic review varied (Supporting File 1: SIGN Quality Checklist). Seven studies were rated as low quality, and the remaining studies were either acceptable (*n* = 3) or high quality (*n* = 2).

The certainty of evidence across outcomes ranged from low to very low (Table 4). Most outcomes were downgraded due to methodological limitations in the included trials, small sample sizes and wide CIs that encompassed both meaningful benefit and no effect. Several outcomes were further downgraded for inconsistency where heterogeneity was moderate and for indirectness where comparator groups included active elements, such as exercise or education. As a result, the certainty of evidence was rated as low for short‐term total PA, short‐term steps and short‐term SB, and very low for all remaining outcomes, including long‐term total PA, MVPA at all time points, longer‐term steps and longer‐term SB.

**TABLE 4:** Summary of findings (GRADE assessment).

Outcome	No. of participants (studies)	Certainty of the evidence (GRADE)	Effect (mean difference, 95% CI)
Total physical activity (min/week) 10–13 weeks	246 (3 RCT studies)	●●○○ Low^a^ ^,^ ^d^	MD: 21.25, 95%CI: −26.11 to 68.61, *p* = 0.38, *I* ^2^ = 46%

Total physical activity (min/week) 12 months	221 (3 RCT studies)	●○○○ Very low^a^ ^,^ ^b^ ^,^ ^d^	MD: 30.52, 95%CI: −25.42 to 86.45, *p* = 0.28, *I* ^2^ = 66%

MVPA (min/week) 10–13 weeks	277 (3 RCT studies)	●○○○ Very low^a^ ^,^ ^b^ ^,^ ^d^	MD: 2.55, 95%CI: −0.66 to 5.75, *p* = 0.12, *I* ^2^ = 37%

MVPA (min/week) 6 months	196 (2 RCT studies)	●○○○ Very low^a^ ^,^ ^b^ ^,^ ^c^ ^,^ ^d^	MD: −11.36, 95%CI: −47.50 to 24.78, *p* = 0.54, *I* ^2^ = 66%

MVPA (min/week) 9–12 months	211 (2 RCT studies)	●○○○ Very low^a^ ^,^ ^b^ ^,^ ^c^ ^,^ ^d^	MD: 2.72, 95%CI: −3.21 to 8.66, *p* = 0.37, *I* ^2^ = 73%

Steps (number of steps/day) 12–14 weeks	167 (3 RCT studies)	●●○○ Low^a^ ^,^ ^d^	MD: 681.35, 95%CI: −143.39 to 1506.09, *p* = 0.11, *I* ^2^ = 0%

Steps (number of steps/day) 3–6 months	190 (2 RCT studies)	●○○○ Very low^a^ ^,^ ^c^ ^,^ ^d^	MD: 703.69, 95%CI: −212.31 to 1619.69, *p* = 0.13, *I* ^2^ = 0%

Sedentary behaviour (min/day) 10–13 weeks	105 (2 RCT studies)	●●○○ Low^a^ ^,^ ^d^	MD: −30.21, 95%CI: −97.56 to 37.14, *p* = 0.38, *I* ^2^ = 0%

Sedentary behaviour (min/day) 6–12 months	98 (2 RCT studies)	●●○○ Low^a^ ^,^ ^d^	MD: −13.27, 95%CI: −86.05 to 59.52, *p* = 0.72, *I* ^2^ = 0%

Abbreviations: CI = confidence interval, MD = mean difference, MVPA = moderate‐to‐vigorous physical activity; RCT = randomised controlled trial.

^a^Downgraded one level due to most studies having unclear or high risk of bias.

^b^Downgraded one level for heterogeneity (large *I* ^2^ and low *p*‐value).

^c^Downgraded one level due to indirectness arising from active control groups that included exercise or education elements.

^d^Downgraded one level for imprecision due to wide CI and low sample size.

## 4. Discussion

OA is a rising public health challenge [43] associated with lower levels of PA [44]. To our knowledge, this is the first systematic review and meta‐analysis to assess the effect of behaviour change combined with exercise interventions on device‐measured PA and SB levels. Overall, there was no evidence that BCTs were effective for increasing the total daily amount of time spent in PA. A meta‐analysis of four studies suggested that behaviour change interventions may result in a small increase in device‐measured MVPA in the short term (10–13 weeks). There was no evidence that BCTs were effective in increasing the amount of time spent in moderate‐to‐vigorous activity in the long term, daily steps or reducing the time spent in SB. Given that most included studies were rated as low quality and the certainty of evidence ranged from low to very low, these findings should be interpreted cautiously. The certainty of evidence ranged from low to very low mainly due to issues of risk of bias (with concealment methods and blinding) and imprecision (to wide CI and small sample sizes). There was high heterogeneity between studies, which likely reflects variation in PA outcomes, intervention characteristics, intervention dosage and duration, delivery mode, BCTs employed, accelerometer protocols, as well as treatment fidelity. These factors, particularly fidelity of the intervention delivery, highlight the need to conduct studies with consistent methodology, to be sure of the true intervention effect on activity behaviours in people with OA.

Current PA recommendations state that older adults, including those with chronic health conditions, such as OA, should take part in 150 min of moderate intensity or 75 min of vigorous intensity PA per week [45]. When assessing the effect of BCTs on the amount of time spent in MVPA, our meta‐analysis found small effect of behaviour change interventions on short‐term device‐measured MVPA and no statistically significant effect on long‐term device‐measured. Although some individual studies reported increases in MVPA, the pooled estimates were associated with wide CIs and moderate‐to‐substantial heterogeneity, indicating considerable uncertainty regarding the effect of these interventions. These findings should be interpreted cautiously given the small number of contributing studies and the low to very low certainty of the evidence. Across the included studies, participants generally accumulated less than the recommended weekly levels of MVPA, regardless of intervention allocation. For example, in two of the three included studies, most participants did not achieve the recommended weekly levels of MVPA, and the increase in MVPA was small (1–2 min/day). In the Gilbert et al. [32] study, the participants with knee OA assigned to the intervention were the only group reported to perform more than 150‐min weekly MVPA but this was only at baseline, while the intervention did not result in any groups of participants achieving the 150‐min MVPA threshold at the follow‐up timepoints, and in fact over the 2‐year follow‐up, recorded an average of 15.85 min per day of MVPA (approximately 40 min per week short of the threshold). Reasons reported by the authors for the nonsignificant change in MVPA include the few number of contact sessions between the therapist and participants and the fact that their participants had a long duration of disease and were mostly overweight or obese [32]. In the Focht et al. [23] study, weekly MVPA levels ranged between 52.4 and 81.1 min from baseline to 12 months, respectively, in the intervention group, which was also classified as mostly having obesity. Only in the Li et al. [39] study, did participants achieve greater volumes of MVPA (on average at least 30 min per day) as measured using the SenseWear Mini. The participants in the latter study were mostly classified as having overweight and reported relatively better levels of symptoms and activities of daily living. Similarly to the findings in our meta‐analysis, in a previous meta‐analysis on the effects of behavioural PA interventions, PA promotion strategies were found to have no overall effect on self‐reported PA levels in people with lower limb OA in the long term [46]. Although the number of studies using device‐based measures of PA included in that meta‐analysis was small (*n* = 3), upon synthesising the data, the authors reported no overall positive effects of the self‐management interventions on device‐based PA [46].

PA is consistently associated with improvements in health outcomes related to people living with OA [47]. However, the current evidence is insufficient to determine whether behaviour change interventions result in meaningful improvements in device‐measured MVPA. The variation in these studies, however, again highlights the need for similar studies to be done to contribute to this field, and there is also a need to address the physical inactivity that is prevalent in this population. Dunlop et al. [4], using data from the Osteoarthritis Initiative, have shown that a minimum threshold of 45 min per week of MVPA predicted improved physical functional outcomes in people with knee OA, a threshold which is consistent across sex, BMI, OA status and age. Further well‐powered studies using consistent accelerometry methods are needed to better understand the effectiveness of these interventions and to identify achievable PA targets for people with OA.

A particular strength of this meta‐analysis is the inclusion of the analysis of SB as an outcome. SB is a distinctly separate behaviour from inactivity as it is defined as low (< 1.5 METs) energy expenditure behaviour during waking hours in sitting and lying postures [48]. People with OA spend much of their waking day in SB [2, 5, 49], which itself is associated with poor health outcomes [50], increasing the risk of comorbidities and resulting in a reduction in physical function [5] and quality of life [51]. For many individuals with OA, increasing light‐intensity activity and reducing sedentary time may represent more achievable behavioural targets than meeting recommended levels of MVPA and may provide functional benefits [52]. However, light PA outcomes were reported inconsistently across studies and using different metrics, preventing meaningful synthesis. Future studies should consider reporting light PA alongside MVPA and SB to provide a more comprehensive understanding of activity behaviour change in this population. In addition, only two studies measured SB as an outcome [36, 39], highlighting the need for studies to measure this behaviour, given its associations with deleterious health effects. Overall behaviour change combined with exercise was shown to have little to no statistically significant difference in SB outcomes in the short and long term. However, the short‐term pooled estimate suggested a reduction of approximately 30 min per day in SB. Although this effect was not statistically significant and the CIs were wide, a reduction of this magnitude may be clinically relevant if sustained over time. Following a 9‐week web‐based behaviour change and PA intervention, Bossen et al. [36] found that the total time in SB decreased over the 12‐month follow‐up period by an average of almost 60 min per day. Similarly, Li et al. [39] reported a reduction in time spent in bouts (≥ 20 min) of SB by over 60 min a day whether participants started their in‐person intervention immediately or after a 14‐week delay. In both studies, the reductions in SB were not statistically significant; however, using data from the NIH‐AARP Diet and Health Study, Matthews and colleagues [53] showed that replacing 1 h of sitting per day with 1 h of light PA in people is associated with a reduction of all‐cause mortality in less active older adults. Thus, future studies using SB change targets for people living with OA may be beneficial to overall health risk.

Although this is the first study to synthesise the evidence for using behaviour change in combination with exercise interventions to modify device‐measured activity behaviours, we found several gaps in the available data for this review. Firstly, the main limitation is that the meta‐analysis was based on a small number of studies (2–3 studies in each analysis) with relatively low participant numbers (98–246 participants). Most of these included studies were rated as low or acceptable quality, and the certainty of evidence was low to very low across outcomes, limiting confidence in the findings. Secondly, there was large heterogeneity in the types of BCTs used as well as method of delivery (four studies delivered the intervention remotely over the phone or web‐based, while the remaining studies delivered the intervention in‐person). There was also considerable variation in the duration of the interventions (14 days–24 months). BCTs were not consistently reported using a standardised framework, such as the Behaviour Change Technique Taxonomy [14], limiting comparison and replication of interventions. In addition, the observed effect of the interventions may be affected by the difference in control conditions used across the studies (three studies used usual care, two studies used a wait list, and one provided education unrelated to PA). For example, some control groups received structured exercise programmes [23, 35, 42] or other forms of PA support [32, 38, 41], which may have reduced the apparent difference between the intervention and control groups. The percentage of variation across the studies varied from *I* ^2^ = 0 to 73%. All these factors contributed to the low certainty of the evidence presented. Additionally, measurement of PA and SB varied in device used, wear position, as well as days worn. Differences in accelerometer models, wear protocols, monitoring duration and outcome definitions may have contributed to measurement variability and reduced comparability between studies. The difference in reporting of outcome measures between studies made further meta‐analysis difficult due to the small number of studies. Only a proportion of studies reported intervention adherence or fidelity, making it difficult to determine whether the intended behaviour change strategies were delivered and received as planned. All included studies were conducted in high‐income countries, which may limit the generalisability of the findings to broader ageing populations and lower‐resource settings. In addition, only English‐language studies were included, and grey literature sources were not systematically searched, which may have increased the risk of language and publication bias.

Behaviour change interventions combined with exercise may support the successful improvement of activity behaviours in those living with OA. However, as highlighted in previous reviews, significant barriers exist to translating research into clinical practice [46]. There remains a need for greater standardisation of research methods in both objective PA measurement and behaviour change interventions. The strength and transferability of studies need to be improved by using defined behaviour change frameworks or theories. In addition, the use of standardised terminology for specific BCTs [14], as well as the inclusion of mediator analysis, will help to identify the active ingredients of the intervention. This consensus will help produce more evidence on the benefits of increasing PA and reducing SB in people with OA. In line with previous reviews, we also highlight the importance of using device‐based measurements of activity behaviours; however, we acknowledge that there is limited consensus in research methodology.

## 5. Conclusion

OA is a common, costly condition associated with pain and decreases in physical function and quality of life [1]. Improving PA levels can enhance OA self‐management, and including BCTs in PA interventions may help support these outcomes in the long term. This systematic review has synthesised evidence for behaviour change combined with exercise intervention to improve activity behaviours measured in a free‐living environment using a device‐based method of measurement in those living with OA. We found some limited evidence that behaviour change and exercise may increase MVPA in the short and long term, but this result should be interpreted with caution due to the lack of data available. This study highlights the ongoing need for methodological rigour in PA measurement, outcome reporting and standardised reporting for BCTs.

## Author Contributions

Rebecca M. Meiring, Estelle D. Watson and Daniel O’Brien: conceptualisation. Mitchell Campbell, Abhishta Sood and Estelle D. Watson: formal analysis and methodology. Rebecca M. Meiring and Estelle D. Watson: writing–original draft. Daniel O’Brien, Mitchell Campbell and Abhishta Sood: writing–review and editing.

## Funding

The study was funded by open access publishing facilitated by the University of Auckland, as part of the Wiley—The University of Auckland agreement via the Council of Australasian University Librarians.

## Conflicts of Interest

The authors declare no conflicts of interest.

## Supporting Information

Additional supporting information can be found online in the Supporting Information section.

## Supporting information


**Supporting Information 1** Supporting File 1: Table of SIGN Quality Checklist.

**Supporting Information 2** Supporting File 2: PRISMA Checklist.

## Data Availability

The data that support the findings of this study are available from the corresponding author upon reasonable request.
