# Supplementary material for: Effects of Behaviour Change Interventions Combined With Exercise on Device‐Measured Physical Activity in People With Hip and Knee Osteoarthritis: A Systematic Review and Meta‐Analysis
Source: J Aging Res. 2026 Aug 17;2026:5852133. doi: 10.1155/jare/5852133 (PMC13479105; doi:10.1155/jare/5852133)
Supplement: Supplementary file 1 [file JARE-2026-5852133-s002.pdf]

Supplementary Table 1. SIGN checklist

Author	year	1	2	3	4	5	6	7	8	9	10	11
Focht et al	2014	yes	can't say	no	no	no	no	yes	3-month: IG =17.5%, CG=22.5%<br>12 month: IG =12.5%, CG= 22.5%.	yes	can't say	-
Bennel et al	2017	yes	yes	yes	yes	no	no	yes	6-month: IG 14.2%, control 16.6%<br>12-month IG 16.6%, control 21.4%<br>18-month: IG 21.4%, control 26.2%	yes	NA	+
Bossen et al	2013	yes	yes	yes	no	no	no	yes	3 month: IG 31.7%, control 23.8%<br>12 month: IG 39%, control 29%	yes	NA	-
Gilbert et al	2018	yes	yes	yes	yes	no	no	yes	6 month: IG 21.4%, control 41.89%	can't say	NA	-
Li et al	2020	yes	yes	yes	no	no	no	yes	13 weeks: IG: 7.69%, control:4%<br>26 weeks: IG 15.38%, control: 8%<br>39 weeks: IG 23.07%, control: 4%	yes	can't say	-
Murphy et al	2018	yes	yes	can't say	yes	yes	yes	yes	IG: n=7 (18%), CG: n=4 (21%)	yes	N/A	++
Murphy et al	2016	yes	yes	can't say	yes	no	no	yes	IG: 22%; CG: 18%	yes	N/A	++
Murphy et al	2012	yes	yes	can't say	no	no	no	yes	6.25%	no	NA	-
Murphy et al	2008	yes	yes	can't say	yes	yes	yes	yes	IG n=3 (10.71%), CG n=0	yes	yes	+
Bell et al	2023	yes	yes	yes	yes	can't say	no	yes	IG n=0, CG n=2 (6%)	no	N/A	+
Christiansen et al	2024	yes	yes	can't say	no	yes	no	yes	14 weeks: IG n=3 (6.5%), CG n=0<br>38 weeks: IG n=5 (total, 10%) (; CG n=0	yes	N/A	-
Gillcrist et al	2024	yes	yes	can't say	can't say	can't say	Can't say	yes	Total sample n=2 (5%)	no	N/A	-

IG = intervention group; CG= control group. Checklist question key. 1: appropriate and clearly focused research question; 2: groups randomised; 3: adequate concealment method used; 4: blinding used; 5: treatment and control groups similar at start of trial; 6: true treatment effect; 7: outcome measures valid and

reliable; 8: drop-out rate; 9: intention to treat analysis; 10: results are comparable between different sites, 11: study quality code: *High quality (++); Acceptable (+); Low quality (-); Unacceptable – reject 0*
